# A New Method of Myostatin Inhibition in Mice via Oral Administration of *Lactobacillus casei* Expressing Modified Myostatin Protein, BLS-M22

**DOI:** 10.3390/ijms23169059

**Published:** 2022-08-13

**Authors:** Dong Kyung Sung, Hyeongseop Kim, Sang Eon Park, Jiwon Lee, Ju-A Kim, Young-Chul Park, Hong Bae Jeon, Jong Wook Chang, Jeehun Lee

**Affiliations:** 1 Department of Pediatrics, Samsung Medical Center, Sungkyunkwan University School of Medicine, Seoul 06351, Korea; 2 Stem Cell Institute, ENCell Co., Ltd., Seoul 06072, Korea; 3 Cell and Gene Therapy Institute, Samsung Medical Center, Seoul 06351, Korea; 4 BL Corporation, Yongin-si 16827, Korea

**Keywords:** myostatin, *Lactobacillus casei*, poly-gamma-glutamic acid synthetase A, Duchenne muscular dystrophy, *mdx* mouse

## Abstract

Myostatin is a member of the transforming growth factor-beta superfamily and is an endogenous negative regulator of muscle growth. This study aimed to determine whether an oral administration of *Lactobacillus casei* expressing modified human myostatin (BLS-M22) could elicit sufficient levels of myostatin-specific antibody and improve the dystrophic features of an animal model of Duchenne muscular dystrophy (DMD; *mdx* mouse). BLS-M22 is a recombinant *L. casei* engineered to harbor the pKV vector and poly-gamma-glutamic acid gene linked to a modified human myostatin gene. Serological analysis showed that anti-myostatin IgG titers were significantly increased, and serum creatine kinase was significantly reduced in the BLS-M22-treated *mdx* mice compared to the control mice. In addition, treatment of BLS-M22 resulted in a significant increase in body weight and motor function (Rotarod behavior test). Histological analysis showed an improvement in the dystrophic features (fibrosis and muscle hypertrophy) of the *mdx* mice with the administration of BLS-M22. The circulating antibodies generated after BLS-M22 oral administration successfully lowered serum myostatin concentration. Myostatin blockade resulted in serological, histological, and functional improvements in *mdx* mice. Overall, the findings suggest the potential of BLS-M22 to treat DMD; however, further clinical trials are essential to ascertain its efficacy and safety in humans.

## 1. Introduction

Duchenne muscular dystrophy (DMD) is a type of muscular dystrophy, and its major symptoms are progressive muscle weakness of the shoulders, arms, respiratory muscles, legs, etc. [1]. DMD is caused by a mutation of the dystrophin gene located on the short arm of the X chromosome (locus *Xp21*), which means that DMD primarily affects males [2]. Dystrophin is one of the important proteins to maintain the cytoskeleton of a muscle fiber [3]. Therefore, the mutation of the dystrophin gene results in extensive muscle deformity, inflammatory responses, and severe fibrosis [4,5,6]. Since DMD patients show low muscle mass and muscle wasting, activating muscle growth or inhibiting negative regulators of muscle growth can be an effective way to cure DMD [7,8].

Myostatin is a member of the transforming growth factor-beta (TGF-β) superfamily and is an endogenous negative regulator of muscle growth [9,10]. Loss-of-function mutations in myostatin result in massive muscle hypertrophy in various species, including humans [10,11]. As the absence of myostatin causes muscle hypertrophy, its therapeutic potential for muscle diseases has been evaluated.

The delivery of antigens by safe, non-invasive vectors, such as commensal lactobacilli, was initially developed using the poly-gamma-glutamic acid synthetase A (pgsA) gene product as an anchoring matrix [12,13]. This antigen delivery system has been applied in treating severe acute respiratory syndrome (SARS) and porcine epidemic diarrhea and the induction of antitumor effects in human papillomavirus type 16 E7-expressing cancer cells [14,15,16]. We hypothesized that myostatin inhibition can be achieved by introducing the modified myostatin antigen linked to an expression vector to enhance mucosal immunity.

To test this hypothesis, we developed a *Lactobacillus casei* expressing modified human myostatin protein (BLS-M22) using the same antigen-presenting platform. This study aimed to address whether an oral administration of BLS-M22 could elicit sufficient levels of myostatin-specific antibodies and whether the generation of the antibodies could improve the dystrophic features of an animal model of DMD (*mdx* model). The study can assist in developing therapeutic strategies to treat DMD, and the application of BLS-22 may be extended to various forms of sarcopenia associated with chronic kidney disease and other chronic illnesses associated with myostatin inhibition.

## 2. Results

### 2.1. Construction of a Novel Surface Display Vector and the Expression of Modified Myostatin Protein on L. casei

**Figure 1:**
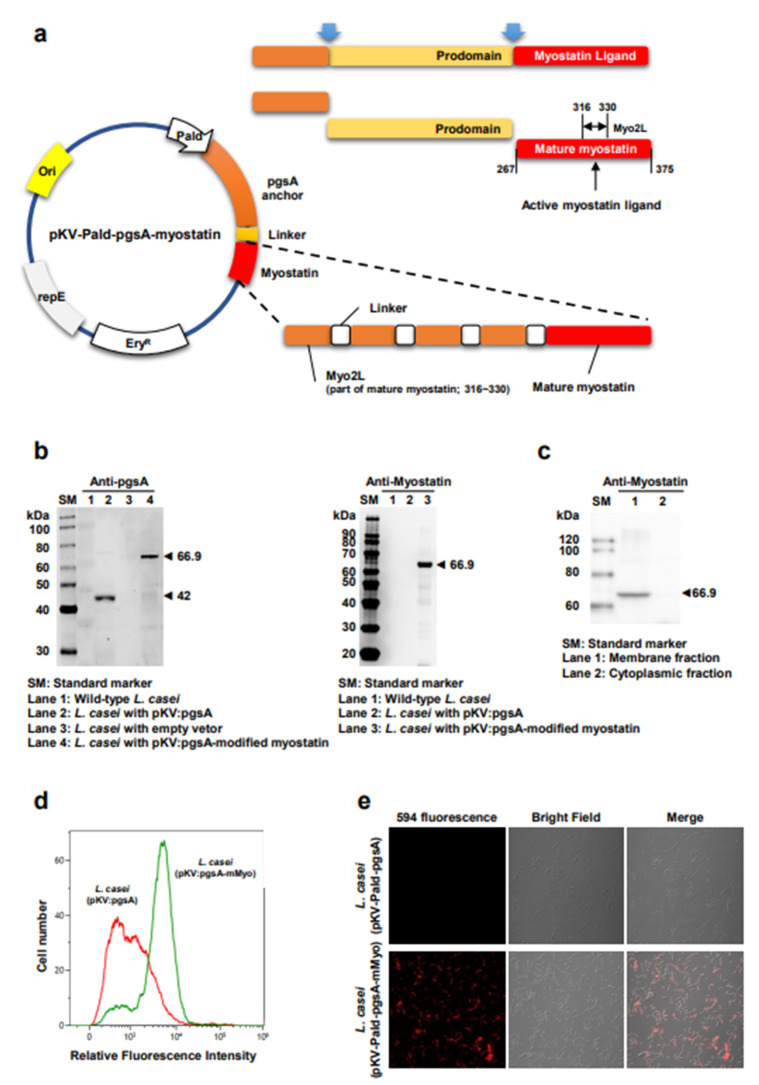
Construction of modified myostatin display vector and confirmation of surface display on *Lactobacillus casei*. (**a**) Schematic diagram for myostatin structure and display vector construction of modified myostatin; (**b**) Western blotting analyses of recombinant modified myostatin (mMyo) expression in *L. casei* using anti-pgsA (**left**) and anti-myostatin (**right**) antibodies. Lanes 1, 2, and 3 show wild-type, recombinant *L. casei* harboring pKV-Pald-pgsA and empty vector, respectively. Lane 4 shows recombinant *L. casei* carrying pKV-Pald-pgsA-mMyo. The expected sizes of pgsA and PgsA-modified myostatin-fused protein are 42 and 66.9 kDa, respectively, SM = standard marker; (**c**) Immunoblotting of cellular fractions of recombinant *L. casei*. Lanes 1 and 2 were loaded with the membrane and the cytoplasmic fractions, respectively. Myostatin fusion proteins were detected in the membrane fraction but not in the cytoplasmic fraction, SM = standard marker; (**d**) Fluorescence-activated cell sorter histograms of recombinant *L. casei* harboring pKV-Pald-pgsA and pKV-Pald-pgsA-mMyo; (**e**) Representative immunofluorescence images of recombinant *L. casei* harboring pKV-Pald-pgsA and PKV-Pald-pgsA-mMyo. Cells were treated with mouse anti-myostatin and Alexa Fluor 594 conjugated anti-mouse IgG antibodies.

We generated expression vectors encoding the pgsA-fused modified myostatin sequence consisting of four repeats of Myo2L, four linkers, and a mature myostatin domain (Figure 1a). Expression of the pgsA-modified myostatin fusion proteins was monitored using immunoblotting whole-cell lysates of serially passaged recombinant *L. casei* (Figure 1b). The pgsA-modified myostatin protein was stably expressed through more than ten serial passages and maintained its predicted molecular mass (66.9 kDa). Further, the membrane and the cytoplasmic fractions of recombinant *L. casei* cells were subjected to immunoblotting. As expected, myostatin fusion proteins were detected in the membrane fraction but not in the cytoplasmic fraction (Figure 1c). Localization of myostatin on the surface of *L. casei* was confirmed using flow cytometry (Figure 1d) and immunofluorescence microscopy (Figure 1e). The results demonstrated the successful expression of modified myostatin on the membrane of *L. casei.*

### 2.2. The Administration Strategy to Achieve the Highest Immunogenicity of BLS-M22 Was Confirmed

**Figure 2:**
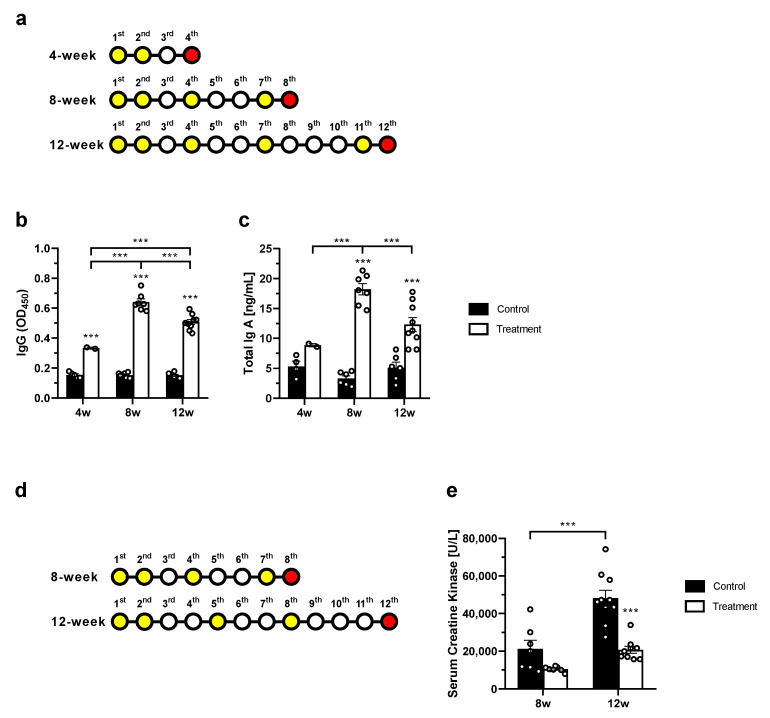
Optimizing administration strategy of BLS-M22 in an *mdx* mouse model. (**a**) Scheme of BLS-M22 administration. The time-points at which the *mdx* mice were administered BLS-M22 (3% of the total feed weight) using a mixed-with-feed method are indicated in yellow circles. The euthanization time-points are shown in red; (**b**) Serum anti-myostatin IgG and (**c**) mucosal total IgA antibody titers after BLS-M22 administration; (**d**) Scheme of BLS-M22 administration for measurement of serum creatine kinase (CK) levels. The week where BLS-M22 (3% of the total feed weight) was administered using a mixed-with-feed method is shown in yellow. The euthanization time-points are shown in red; (**e**) Serum CK levels after BLS-M22 administration. All data are presented as mean ± standard error of the mean (SEM). *** *p* < 0.001.

To assess the immunogenicity of BLS-M22, the *mdx* mice were administered BLS-M22 via a mixed-with-feed method with 3% of the total feed weight. Then the serum anti-myostatin IgG antibody and the intestinal IgA antibody titers were monitored (Figure 2). The *mdx* mice in the control and treatment groups were assigned to three experimental subgroups based on the duration of BLS-M22 administration—4-, 8-, and 12-week subgroups where BLS-M22 was administered 2, 4, and 5 times, respectively (Figure 2a). In the treatment group, the serum anti-myostatin IgG increased significantly in the 8-week subgroup (0.64 ± 0.06) compared to that in the 4-week subgroup (0.33 ± 0.01; *p* < 0.01), which was further reduced in the 12-week subgroup (0.51 ± 0.05; *p* < 0.01). The mucosal total IgA antibody titer showed a similar trend in the three subgroups (8.86 ± 0.37, 18.23 ± 2.51, and 12.33 ± 3.51, respectively); however, the difference between the 4- and 12-week subgroups was not significant (Figure 2b,c). In contrast, in the control group, no significant difference was observed in the serum anti-myostatin IgG and mucosal total IgA antibody titer between the three subgroups (Figure 2b,c). Nevertheless, the titers of serum anti-myostatin IgG antibody and mucosal total IgA antibody in the intestinal lavage fluid were significantly higher in all subgroups of the treatment group than those in the control group (*p* < 0.001 at all three time-points, Figure 2b,c).

Next, serum creatine kinase (CK) level (Figure 2e) was measured to identify the lasting period of therapeutic effect under BLS-M22 administration. The serum CK level of control *mdx* mice at 8 weeks was 21,257 ± 11,923 U/L, which increased to 48,160 ± 13,417 U/L at 12 weeks (*p* < 0.001) as expected. On the contrary, BLS-M22 administration decreased serum CK levels at both 8 weeks of treatment (10,514 ± 1362 U/L) and 12 weeks of treatment (20,732 ± 5693 U/L). Especially, a significant decrease in serum CK was observed in 12-week treatment mice (*p* < 0.001 vs. 12-week of control). These results indicated that BLS-M22 administrated four times was the best way to achieve the highest production of anti-myostatin antibodies. At least 12 weeks were needed to observe significant physiological changes induced by BLS-M22.

### 2.3. BLS-M22 Promoted Body Weight Gain and Physical Activity

**Figure 3:**
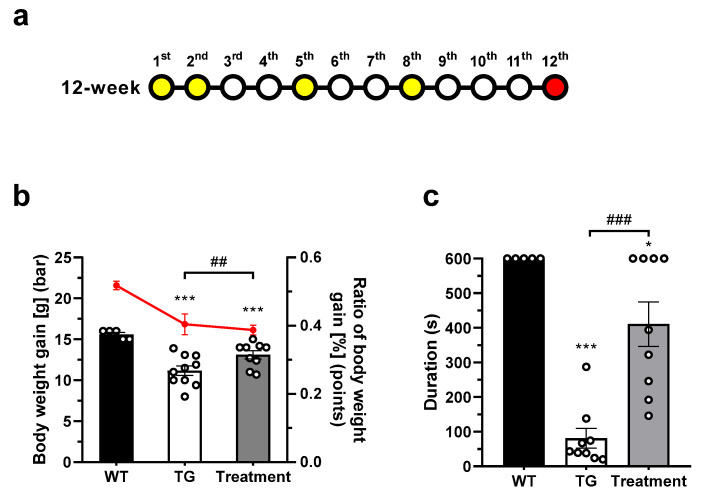
Measuring changes in body weight and duration time (rotarod test) followed BLS-M22 administration in the *mdx* mouse model. (**a**) Schematic diagram of BLS-M22 administration schedule. The weeks on which *mdx* mice were treated with BLS-M22 via a mixed-with-feed method with 3% of the total feed weight are indicated in yellow. The euthanization time-points were in the red; (**b**) The body weight gain (bar graph) and body weight gain ratio (line graph in red) at 12 weeks. (**c**) Rotarod behavior test was performed, and the length of time (s) the mice were able to maintain their balance was recorded (duration time). WT, wild-type group; TG, transgenic *mdx* group; Treatment, BLS-M22 treated group. All data are represented as mean ± SEM. * *p* < 0.05 and *** *p* < 0.001 vs. WT; ## *p* < 0.01, and ### *p* < 0.001 vs. TG.

To assess the therapeutic effect of BLS-M22, we observed physical change in wild-type (no treatment) and *mdx* mice under the optimized administration schedule (Figure 3a). We calculated and compared the body weight gain and the body weight gain ratio among the three experimental groups (Figure 3b). The body weight gain in wild-type (WT) mice (15.60 ± 0.55 g) was significantly higher than that in transgenic *mdx* (TG; 11.18 ± 1.86 g) and BLS-M22 treated mice (Treatment; 13.11 ± 1.50 g). Moreover, the body weight gain in Treatment mice was significantly higher than that in TG (*p* < 0.01). The body weight ratio also showed a similar trend; it was higher in WT mice (0.52 ± 0.03%) than those in TG (0.40 ± 0.09%) and Treatment mice (0.39 ± 0.04%). However, the differences in the body weight gain ratio of the three experimental groups were not statistically significant.

Next, behavioral changes were observed using the rotarod test (Figure 3c). In WT mice with no physical disability, the duration for which the mice were able to maintain balanced walking was 600 s. However, the duration reduced to 81.11 ± 85.12 s in TG mice, indicating strong dystrophy. On the contrary, the duration of balanced walking was significantly improved (411.1 ± 192.5 s) in BLS-M22 administered mice (*p* < 0.05 vs. WT, and *p* < 0.001 vs. TG).

### 2.4. BLS-M22 Reduced Fibrosis and Hypertrophy in Lower Extremity Muscle

**Figure 4:**
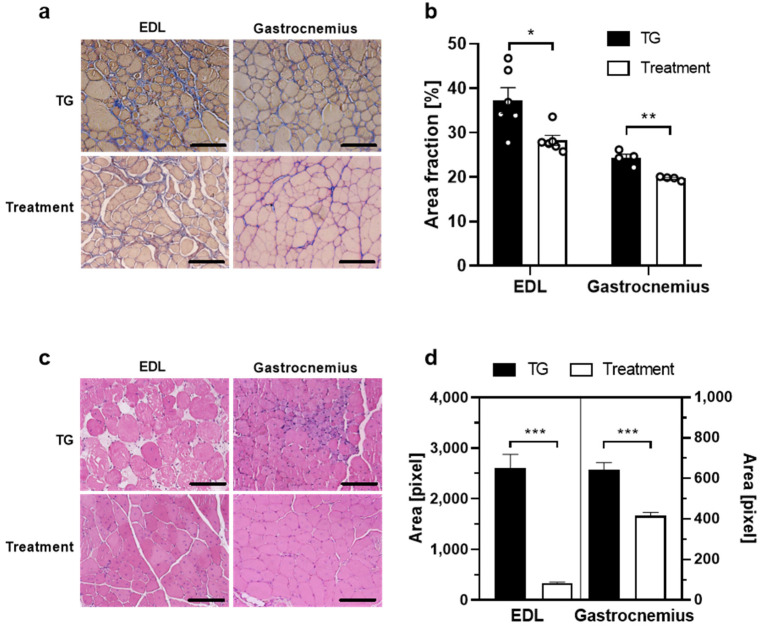
Histological assessment after BLS-M22 administration in the *mdx* mouse model. (**a**) Masson’s trichrome staining images. The fibrosis area is stained in blue; (**b**) Fibrosis area fraction was quantified from each microscopic image. * *p* < 0.05, ** *p* < 0.01; (**c**) Hematoxylin and eosin staining images of cross-sectional muscle tissue paraffin slides; (**d**) The cross-sectional area was measured using Image J software. The cross-sectional area of the extensor digitorum longus (EDL) muscle is shown on the left y-axis, and that of the gastrocnemius muscle is on the right y-axis. Scale bar in (**a**,**c**): 100 μm. All data are presented as mean ± SEM. *** *p* < 0.001.

Histological analysis was conducted to confirm the therapeutic effect of BLS-M22. First, BLS-M22 was administered according to the optimized schedule (Figure 3a), and extensor digitorum longus (EDL) and gastrocnemius muscle were harvested at 12 weeks. Masson’s trichrome staining (Figure 4a) and the quantification of the fibrosis area from the microscopic images (Figure 4b) revealed the degree of fibrosis in muscle tissue. In EDL, the fibrosis area fraction in the mdx control mice (TG) was 37.29 ± 7.05%, which significantly decreased to 28.30 ± 2.73% in the BLS-M22-administered mice (*p* < 0.05; Figure 4b. In addition, the fibrosis area fraction in the gastrocnemius muscle of the TG group (24.35 ± 1.65%) was significantly higher than that in the gastrocnemius muscle of the treatment group (19.73 ± 0.44%) (*p* < 0.01; Figure 4b).

Next, H&E staining images were obtained to observe hypertrophic muscle fibers in each muscle tissue (Figure 4c), and the cross-section area (CSA) of the muscle fiber was quantified (Figure 4d). In EDL muscles of the TG mice, the average CSA was 2613 ± 1773 pixels, which was significantly decreased to 339.9 ± 161.8 pixels in BLS-M22 administered mice (*p* < 0.001). Similarly, the average CSA in the gastrocnemius muscle of the TG group (642.9 ± 403.8 pixels) was significantly decreased in the treatment group (415.3 ± 146.3 pixels; *p* < 0.001; Figure 4d).

## 3. Discussion

This study showed that *L. casei* expressing modified myostatin protein on its surface (BLS-M22) inhibited myostatin and improved the dystrophic features of *mdx* mice. BLS-M22 enhanced mucosal immunity and produced systemic anti-myostatin IgG antibodies, which consequently reduced the serum CK. Furthermore, BLS-M22 increased the body weight of *mdx* mice and improved physical activity (rotarod behavior test). BLS-M22 also diminished the extent of deposition of fibrous muscle tissue and hypertrophic muscle fibers. These results prove that the modified myostatin linked to the pgsA anchor on the surface of *L. casei* can act as an effective tool to restore muscle damage while inhibiting myostatin.

The expression of antigens on the bacterial surface is difficult because large-sized antigens can disturb the cell membrane topology [15]. In addition, the average thickness of the peptidoglycan layer in *L. casei* is approximately 15–30 nm [17]; hence, it requires a transmembrane anchor, which can cross the cell wall. In this study, a novel expression vector, pgsA gene product, was used as an anchoring matrix. PgsA is a transmembrane protein derived from the poly-gamma-glutamic acid synthetase complex of *Bacillus subtilis* [12,13]. It has one transmembrane domain near its N-terminus (amino acids 26 to 42), with the bulk of the protein (approximately 336 amino acids) located outside the cell membrane [13,15]. It has been proven safe and effective for generating mucosal and systemic antibodies [14,15,16]. The first trial using this antigen presentation platform was conducted for the severe acute respiratory syndrome-associated coronavirus (SARS-CoV) [15]. In their study, Lee et al. demonstrated that intranasal and oral vaccination of the mice with the recombinant *L. casei* elicited systemic and mucosal immune responses that had potent neutralizing activities against the SARS pseudovirus [15]. Using the same antigen-presenting platform, myostatin immunization was performed in normal mice and chicken [18], where *L. casei* expressing the mature domain of avian myostatin on its cell surface was produced. In that study, oral inoculation led to an antibody response against the avian myostatin and increased body weight in normal mice and chickens. These results suggest the potential of avian myostatin to increase body weight and skeletal muscle mass [18]. On the contrary, in this study, we used *L. casei* expressing modified myostatin (BLS-M22) to inhibit myostatin and cure DMD. Here, we hypothesized that myostatin inhibition by BLS-M22 induces muscle growth and reduces the dystrophic feature of DMD. Therefore, in this study, we used the *mdx* mouse model, which is a well-known in vivo model of DMD.

The first preclinical trial of myostatin blockade to treat the DMD in a mouse model (*mdx* mouse) was performed using a monoclonal antibody against myostatin [19]. The study showed that myostatin blockade resulted in the functional improvement of the dystrophic phenotype as assessed by anatomical, physiological, and biochemical parameters. Myostatin inhibition was also attempted with adeno-associated virus serotype 8 (AAV8) vectors delivering the myostatin propeptide gene [20]. This study by Qiao et al. showed that intravenous injection of AAV8 carrying a modified propeptide fused with the IgG Fc gene in wild-type adult mice enhanced muscle growth through myofiber hypertrophy. In addition, the treated *mdx* mice showed larger and more uniform myofibers, fewer infiltrating mononuclear cells, less fibrosis, and lower serum CK.

Based on the therapeutic effect of myostatin inhibition, a phase I/II human clinical trial of a myostatin inhibitor, MYO-029, was also performed [21]. The clinical study was performed on adult patients with Becker muscular dystrophy, facioscapulohumeral dystrophy, and limb-girdle muscular dystrophy. The study revealed that MYO-029 is safe, with no significant adverse effects. Although no improvement in muscle strength was observed in the trial, increased muscle mass and myofiber diameters were speculated to be due to the biological activity of MYO-029. In addition, six randomized, double-blinded, placebo-controlled trials of various myostatin inhibitors in humans have been conducted. However, these clinical trials did not show sufficient efficacy of MYO-029 in treating muscular dystrophy [21,22].

To overcome the failure of clinical trials with a myostatin inhibitor using a monoclonal antibody, four repeats of the myostatin active domain segment protein were fused to pgsA as displayed on the surface of *L. casei* (Figure 1). The amino acid sequence of the myostatin antigenic moiety used in this study was the same as that of human myostatin. The amino acid sequence of the active myostatin, the proteolytically processed carboxy-terminal site, has 100% homology among the rat, mouse, pig, chicken, and human proteins, suggesting a common and highly conserved function [23]. It could be atypical that the human immune system responds to self-antigen. In this study, BLS-M22 harbors modification of the myostatin protein, which is postulated to enhance immunity to the self-antigen, myostatin itself. The results of this study support this hypothesis and provide evidence that the transformation of the myostatin protein produces an immune reaction to the original myostatin protein.

Generally, this type of vaccination first induces mucosal immunity [15]. Extracellularly accessible antigens expressed on the surface of bacteria are better recognized by the immune system than intracellular antigens [24]. The production of circulatory IgG and mucosal IgA antibodies has been proven in a previous study with a similar design [15,25]. In this study, total mucosal IgA and serum anti-myostatin IgG antibody titers increased significantly. From the fourth week, the presence of a specific serum IgG antibody was confirmed. It increased two-fold in the 8th week and remained by the 12th week with booster doses.

In this study, BLS-M22 was administered to mice via a mixed-with-feed method. There are pros and cons to the mixed-with-feed method. First, no special skill is required to administer BLS-M22 to mice when using the mixed-with-feed method, and it is a more ethical method. Second, BLS-M22 can be taken all day long; therefore, the immune response can remain relatively stable. However, since the mixed-with-feed method relies entirely on mouse intake, it is difficult to determine the exact amount of administration, and it can show high individual differences. To overcome those disadvantages of the mixed-with-feed method and acquire evidence for clinical application of the myostatin inhibition effect of BLS-M22, we administered 30 mg or 60 mg BLS-M22 orally using an oral zoned needle (Figure S1). The serum IgG antibody was persistently present until the 20th week following BLS-M22 administration with the once-daily oral gavage method (Figure S1b), and serum myostatin concentration was efficiently inhibited according to the IgG production in dose-dependent manners (Figure S1c). Therefore, it is essential to determine the optimal dose of *L. casei* presenting the modified myostatin to ensure an adequate anti-myostatin antibody titer.

Not only the dose of *L. casei* expressing the modified myostatin, but the optimal administration strategy is also important to achieve the highest therapeutic efficacy. In Figure 2, we optimized the frequency of BLS-M22 administration and estimated the duration required to show a significant reduction in serum CK in the *mdx* mouse. We intentionally adjusted the administration interval of the BLS-M22 widely because of its expected mechanism of action. Moreover, maintaining the anti-myostatin antibody levels high but not saturated is essential for realizing the potential therapeutic effect of BLS-M22 as it depends on the immune response of an individual toward BLS-M22. The findings of this study demonstrated that the serum anti-myostatin IgG was the highest when BLS-M22 was administered for 12 weeks to *mdx* mice; however, this might not be the most effective way. We also showed that excessive administration could decrease anti-myostatin IgG and total IgA production (Figure 2a–c). From this result, we decided to treat BLS-M22 with some intervals to evade saturation and even a decline in the efficiency of antibody production (Figure 2d). Although we had not compared the number of all cases, our results indicated that administering BLS-M22 at a moderate level would be effective. Nevertheless, it is necessary to establish an efficient optimal administration strategy for clinical applications.

Myostatin is a member of the TGF-β family of signaling molecules, and the blockade of myostatin causes muscle cell growth and increases muscle power [9,10,26]. In this study, blockade of myostatin resulted in a significant increase in the single fiber area in the treated *mdx* mouse group. The treated *mdx* mice showed an increase in the total body weight and performed better on the rotarod test than the control *mdx* mice, implying improved gross motor function with BLS-M22 administration. Based on these results, the increase in body weight could be attributed to the growth of myofibers resulting from the inhibition of myostatin by BLS-M22.

Furthermore, blocking of myostatin may permit hypertrophic signals to drive muscle growth above the point where it is compromised by normal workloads, resulting in a shift of dystrophic muscles above the damaging stress levels as previously suggested by Zammit and Partridge [27]. Barton et al. suggested that having a greater functional mass with muscle hypertrophy could protect against damage [28]. The serum CK level in the treated *mdx* mice decreased to half of that in the control *mdx* mice. Elevated CK concentrations have been consistently observed in *mdx* mice and DMD patients owing to sarcolemmal damage [29]. According to a previous study, the muscles of *mdx* mice treated with the monoclonal antibody showed improvements in physical performance and force generation as well as a decrease in serum CK levels to near wild-type levels [19]. Furthermore, the inhibition of myostatin renders most dystrophin-deficient muscles resistant to necrosis during normal daily activities [19,27]. In our study, compared to *mdx* mice, the treated mice showed profound improvements in dystrophic features, a decrease in fibrosis, and muscle hypertrophy. These results suggest that the inhibition of myostatin ameliorates dystrophic features.

There are several points to consider in terms of the clinical application of this antigen-presenting system. At least six molecules (MYO-029, BMS-986089, PF-06252616, ACE-083/-031, BYM338, and FS-344) have been developed by pharmaceutical companies to inhibit myostatin pathways [30]. These molecules have been evaluated for their efficacy and safety in various neuromuscular diseases. However, these studies have not shown significant improvements in motor power during human clinical studies [21,30,31,32]. Regarding the discrepancy between preclinical animal studies and human clinical studies, three possibilities have been suggested. First, the pharmacokinetics/pharmacodynamics of the experimental molecules differed between animal models and human subjects. Second, there is a lack of specificity of the drugs in inhibiting the myostatin pathways for GDF8, GDF11, and ACTIVIN A; therefore, inhibition was insufficient. Third, a low expression level of myostatin in neuromuscular diseases was detected, which translates to the loss of the target for myostatin pathway inhibition [30]. Regarding the low expression level of myostatin, the study showed a two-fold or higher decrease in circulating myostatin in the most atrophic disease, spinal muscular atrophy (SMA), and the most wasting disease, DMD. Becker muscular dystrophy, inclusion body myositis, and facioscapulohumeral muscular dystrophy patients with less prominent muscle atrophy show higher circulating myostatin concentrations than DMD and SMA patients but lower levels than normal controls [30]. Based on the third explanation, Mariot et al. showed that the restoration of *Mtm1* expression in the congenital myotubular myopathy mouse model (knockout of *Mtm1*) is associated with a normalization of the myostatin pathway, which implies that restoration of the dystrophin protein might be necessary for myostatin inhibition. The downregulation of the myostatin pathway in neuromuscular disease explains the ineffectiveness of previous clinical trials. It also provides solutions for using myostatin inhibition methods in neuromuscular diseases. Therefore, if myostatin inhibition treatment is applied with gene-restoring therapy such as antisense oligonucleotide or gene correction with virus vector, the therapeutic effect will be synergistic.

Supporting studies have shown more potent effects of myostatin pathway inhibition therapy in *mdx* mice with dystrophin restoration through exon skipping [33,34,35]. Recently, combination treatment with myostatin inhibition and antisense oligonucleotide therapy has improved SMA outcomes [36]. Considering these results, we speculate that myostatin inhibition through a surface antigen-presenting system using *L. casei* could have a more promising therapeutic effect if used with dystrophin restoring therapy, such as antisense oligonucleotide therapy, mini-dystrophin gene delivery via viral vectors, or the read-through method. Furthermore, myostatin inhibition has been studied in various forms of sarcopenia associated with chronic kidney disease and other chronic illnesses [37,38]. BLS-M22 may be considered for these conditions, besides genetic muscle diseases, after further preclinical validation.

## 4. Materials and Methods

### 4.1. Bacterial Strains, Cloning, and Construction of Surface Expressing Plasmids

To express antigens on the surface of *L. casei*, a pKV vector containing a highly constitutive promoter (the aldolase promoter from *L. casei*) and pgsA (derived from *Bacillus subtilis*) was used as an anchor. The modified myostatin sequence comprised four repeats of Myo2L, four linkers, and a mature myostatin domain. Myo2L is a specific oligopeptide region comprising the amino acid residue from 316 to 330 of the mature myostatin (myostatin (*Homo sapiens*); GenBank: ABI48422.1) and is expected to have high antigenicity. The 615 bp DNA fragment encoding the modified myostatin was chemically synthesized considering codon usage for *L. casei* and confirmed by sequencing analysis (Cosmogenetech, Seoul, Korea). The pgsA-modified myostatin-fused recombinant protein expression vector was constructed by inserting the modified myostatin sequence into the pgsA C-terminal region of the pKV vector expressing the pgsA anchor protein using the BamHI and XbaI restriction enzymes. The vectors were first established in *Escherichia coli* DH5α and then transformed into *L. casei* via electroporation. Recombinant *L. casei* cells were grown in De Man, Rogosa, and Sharpe medium with erythromycin (16 μg/mL) at 30 °C. *E. coli* DH5α strain was grown in Luria–Bertani medium with erythromycin (150 μg/mL) at 37 °C.

### 4.2. Western Blot Analysis

Recombinant *L. casei* cells were harvested, washed three times with 700 μL phosphate-buffered saline (PBS), resuspended in 500 μL PBS containing 1 mM phenylmethylsulfonyl fluoride, and sonicated. The samples were placed on ice (all subsequent steps were performed on ice), and 100 μL of the cell lysate was mixed with 30 μL of 5× sample buffer and boiled for 10 min. The samples were centrifuged at 16,000× *g* for 3 min at 4 °C, and the supernatants were resolved using 12% sodium dodecyl sulfate-polyacrylamide gel electrophoresis and transferred to polyvinylidene difluoride membranes (Millipore, Burlington, MA, USA). The pgsA-modified myostatin fusion protein bands were detected by labeling with rabbit anti-pgsA IgG antibody, which was a custom-made (AbClone, Seoul, Korea) polyclonal antibody raised against pgsA protein (domain 2 and 3), and mouse anti-myostatin IgG monoclonal antibody (ab201954, Abcam, Cambridge, UK), respectively. Subsequently, horseradish peroxidase (HRP)-conjugated goat anti-rabbit IgG antibody and anti-mouse IgG antibody, diluted in 5% skim milk, were added, respectively. The blots were developed using an enhanced chemiluminescence detection kit (Amersham Biosciences, Uppsala, Sweden) and detected using a luminescence analyzer (Fujifilm LAS-3000, Tokyo, Japan).

The membrane and the cytoplasmic fractions of recombinant *L. casei* cells were subjected to immunoblotting to determine the cellular localization of the myostatin fusion proteins. Briefly, the cell pellet was resuspended in the lysate buffer, PBS supplemented with 5 mM ethylenediaminetetraacetic acid (EDTA) and 1 mM phenylmethylsulfonyl fluoride (PMSF), and transferred to the tubes in which the zirconia beads (0.1 mm in diameter; KT03961-1-304.7; Bertin instruments; Montigny-le-Bretonneux, France) were added. The tubes were loaded onto the homogenizer (Precellys Evolution, Bertin instruments) and homogenized at 6800 rpm for 30 s, followed by centrifugation (3000 rpm; 4 ℃; 1 min). The lysate supernatants were collected and centrifuged at 7000 rpm for 10 min at 4 °C. The supernatants were centrifuged at 16,000× *g* for 2 h at 4 °C. Finally, the supernatants (cellular fraction) and the pellets (membrane fraction) dissolved in the lysate buffer were used for immunoblotting as described elsewhere.

### 4.3. Flow Cytometric Analysis and Immunofluorescence Microscopy

For flow cytometric analysis, *L. casei* cells were harvested, washed twice with PBS, and resuspended in Tris-EDTA (TE) buffer containing mouse anti-myostatin antibody (ab201954, Abcam Cambridge, UK). The cell suspensions were washed twice with PBS and then incubated on ice for 2 h with fluorescein isothiocyanate-conjugated anti-mouse IgG antibody (Molecular Probes, Eugene, OR, USA) in TE buffer. The resulting cell pellets were resuspended in PBS and assayed with a Navios flow cytometer using the Kaluza software (Beckman Coulter, Brea, CA, USA). For immunofluorescence, cells labeled with mouse anti-myostatin antibody (ab201954, Abcam, Cambridge, UK) and Alexa Fluor^®^ 594-conjugated anti-mouse IgG antibody (Molecular Probes, Eugene, OR, USA) were examined using a fluorescence microscope (Axioskop 2, Carl Zeiss, Oberkochen, Germany) and photographed using an Axiocam HR camera (Carl Zeiss, Oberkochen, Germany). The exposure times were identical.

### 4.4. Animal

This study was reviewed and approved by the Institutional Animal Care and Use Committee of the Samsung Biomedical Research Institute (SBRI; approval number: C-B1-309-1). The SBRI is an Association for Assessment and Accreditation of Laboratory Animal Care International-accredited facility and abides by the Institute of Laboratory Animal Resources guide.

Male and female mdx mice (C57BL/10ScSn-Dmd^mdx^/J) and normal control mice (C58BL/10ScSn J) were purchased from The Jackson Laboratory (Bar Harbor, ME, USA). Male mdx mice were selected and confirmed as transgenic mice through genotyping according to the method recommended by The Jackson Laboratory (Protocol 21940). Only male mdx mice were enrolled in all experiments.

### 4.5. Administration of BLS-M22

BLS-M22 (BL Corporation, Yongin-si, Korea) for the treated group and *L. casei*–pgsA (*L. casei* carrying pgsA, but no myostatin) for the control or normal group were prepared in a heat-inactivated and lyophilized powder form. Briefly, the seed culture of BLS-M22 in MRS broth was supplemented with erythromycin (16 µg/mL) for 18 h at 30 °C in a 1.5 L fermenter until reaching an optical density (OD) of 6.0 ± 1.0 at 600 nm (OD_600_), followed by the primary culture in a 50 L fermenter for 18–20 h to obtain an OD_600_ of 9.0 ± 1.0. After cooling down to 10 °C, maltose was added to the culture medium to a final concentration of 5% and then kept for 2 h to stabilize the cells during heat inactivation. Subsequently, the heat inactivation of the recombinant *L. casei* was achieved by a gradual increase in temperature (60, 80, 85, and 90 °C) with a cooling step down to 30 °C between temperatures. This process resulted in the complete inactivation and maintenance of the unharmed surface antigens. The cell pellets recovered by centrifugation at 12,000× *g* and 4 °C for 9 min were washed three times with sterile distilled water and then freeze-dried. Lyophilized recombinant *L. casei* was collected in a sterile aluminum bag and stored at 4 °C until use. The concentration of *L. casei* was 1.2 × 10^9^ colony-forming units per gram. It was delivered using a mixed-with-feed method (3% of BLS-M22 for the treated group and 3% of *L. casei*-pgsA for the control group mixed with 97% of mouse feed). The mice (6 weeks old) were divided into control and treatment groups and fed the *L. casei*-pgsA or BLS-M22, respectively. The initial doses were administered in the first 2 weeks (6 and 7 weeks of age), and booster doses were administered at designated time points according to the design of the experiment (Figure 2 and Figure 3). In Figure S1, the control mdx group was orally administered with 100 μL saline, and the treatment group was orally administered with 60 mg of BLS-M22, respectively, diluted in 100 μL of saline using an oral Zonde needle (20-gauge diameter, Jeung Do BIO & PLANT) connected to a syringe.

### 4.6. Physical Measurement (Body Weight), Behavioral Evaluation (Rotarod Test)

The body weight of each mouse was measured every week. The weight gain during the study period (final body weight—initial body weight) was used to compare the differences between the groups.

The rotarod apparatus (Samkwang, Korea) was used for the functional evaluation of the mice. The rotation speeds were 15 rpm. Each mouse had six training sessions per day for three days during the week before the sacrifice. The maximum value among the three replicates of the trial was selected as the final record.

### 4.7. Blood Collection

The mice were sacrificed under general anesthesia with ketamine (0.1–0.5 mg/kg via intraperitoneal injection until full sedation). Whole blood was collected by direct puncture of the left ventricle. The serum was separated by centrifugation at 2000× *g* for 5 min and stored at −70 °C until used for measurements of anti-myostatin IgG, myostatin, or serum creatine kinase (CK) concentrations.

### 4.8. Serum Creatine Kinase Assay

Whole blood samples obtained after sacrifice at the end of the experiment were subjected to serum CK measurements. Serum CK was quantitated using an indirect CK colorimetric assay kit and associated standards (Sigma-Aldrich, St. Louis, MO, USA), following the instructions available with the kit.

### 4.9. Quantitation of Anti-Myostatin IgG Antibody in Serum

Serum samples obtained from the animals were assessed to measure the myostatin-specific IgG antibody generated in response to treatment with BLS-M22. Myostatin (R&D Systems, Minneapolis, MN, USA) was diluted to 1 µg/mL with 50 mM carbonate buffer (pH 9.0, Sigma-Aldrich). The diluted myostatin (50 µL/well) was infused into 96-well plates and allowed to coat the wells with gentle shaking overnight. After three cycles of washing with PBS, 200 µL PBS with 1% bovine serum albumin was added to each well and incubated for 1 h at room temperature. Again, after three rounds of washing, 100 µL of experimental serum (10 µL/mL) was added to each well and incubated for 1 h at room temperature. Following another three cycles of washing, PBS containing 0.05% Tween 20 and 100 µL diluted HRP-conjugated anti-mouse antibody (1:1000) was added to each well. After 1 h of incubation, three final rounds of washing were performed with PBS containing 0.05% Tween 20 and 50 µL 3,3′,5,5′-Tetramethylbenzidine (TMB) substrate solution (Sigma-Aldrich) was added to each well. The samples were incubated until color appeared, after which 50 µL stop solution was added, and the samples were read using a microplate reader (Bio-Rad) at 450 nm.

### 4.10. Tissue Harvest and Histological Analysis

Individual muscle tissues (gastrocnemius and EDL muscles) were collected following cardiac perfusion with normal saline and fixed in formalin solution. The muscle samples were prepared in paraffin blocks. Serial sections (4 µm thick) were cut and stained with hematoxylin and eosin (H&E). Images were taken using an Olympus IX51 inverted microscope (Tokyo, Japan). Morphometric measurements and analyses were performed with the digital images using the ImageJ software (version 1.51, National Institutes of Health, Bethesda, MD, USA).

For the fibrosis measurement, paraffin sections of the gastrocnemius and EDL muscle sections were deparaffinized, rehydrated, and then stained using the Masson’s Trichrome Stain Kit (Sigma-Aldrich, St. Louis, MO, USA), according to the manufacturer’s instructions. The percentage of Masson staining positive area was determined, and Image J was used for quantitative analysis.

### 4.11. Intestinal Lavage Fluids Harvest and Measurement of Total Mucosal IgA Antibody

Intestinal lavage fluid was collected from the mice by washing the terminal part of the small bowel (the whole ileum and part of the jejunum; total length of 30 cm). The washing was performed using 1 mL Dulbecco’s PBS (Welgene, Gyeongsan-si, Korea) supplemented with a protease inhibitor cocktail (Roche Diagnostics, Mannheim, Germany). The lavage samples were centrifuged for 20 min at 12,000× *g*; at 4 °C. The supernatants were stored at −70 °C.

Intestinal lavage fluid samples collected from the mice were submitted to quantify total intestinal IgA using a commercial total IgA Mouse enzyme-linked immunosorbent assay (ELISA) kit (Abcam, Cambridge, MA, USA) according to the manufacturer’s protocol. The samples were read under a microplate reader (Bio-Rad, Hercules, CA, USA) at 450 nm.

### 4.12. Enzyme-Linked Immunosorbent Assay (Myostatin)

Whole blood was extracted by cardiac puncture and collected using an EDTA tube. Within 60 min of collection, the samples were centrifuged at 1000× *g* for 15 min, and the plasma samples (supernatants) were stored at −80 °C. The concentration of myostatin was measured in plasma using an ELISA Kit according to the manufacturer’s protocol (R&D Systems). The OD was measured using a microplate reader (Bio-Rad).

### 4.13. Statistical Analysis

Prism version 8.3.1 (GraphPad Software, San Diego, CA, USA) was used for statistical analyses. Two-way ANOVA with Tukey’s multiple comparison test was used to compare two variables between the three groups. Unpaired two-tailed Student’s *t*-test was performed to determine statistical significance between the two experimental groups.

## 5. Conclusions

In conclusion, BLS-M22, which was produced via the expression of the myostatin antigenic moiety conjoined with the pgsA vector to the surface of *L. casei*, successfully blocked myostatin by generating circulatory anti-myostatin antibodies in *mdx* mice via the mixed-with-feed method (3% of BLS-M22 for the treated and 97% of mouse feed). Myostatin blockade resulted in physiological (serum creatine kinase level), physical (body weight change), functional (rotarod test), and histological (fibrosis, cross-sectional area of EDL, and gastrocnemius) improvements in the dystrophic features of *mdx* mice. The findings suggest that BLS-M22 treatment can be applied to human DMD after verifying its efficacy, safety, and optimal dose. It may also be applied to various muscle diseases and sarcopenia associated with chronic conditions.

## Data Availability

Not applicable.

## Supplementary Materials

The following supporting information can be downloaded at: https://www.mdpi.com/article/10.3390/ijms23169059/s1.

## References

[1] Duan D., Goemans N., Takeda S., Mercuri E., Aartsma-Rus A. (2021). Duchenne muscular dystrophy. Nat. Rev. Dis. Primers.

[2] Moser H. (1984). Duchenne muscular dystrophy: Pathogenetic aspects and genetic prevention. Hum. Genet..

[3] Blake D.J., Weir A., Newey S.E., Davies K.E. (2002). Function and Genetics of Dystrophin and Dystrophin-Related Proteins in Muscle. Physiol. Rev..

[4] Kharraz Y., Guerra J., Pessina P., Serrano A.L., Muñoz-Cánoves P. (2014). Understanding the Process of Fibrosis in Duchenne Muscular Dystrophy. BioMed. Res. Int..

[5] Monici M.C., Aguennouz M., Mazzeo A., Messina C., Vita G. (2003). Activation of nuclear factor-kappaB in inflammatory myopathies and Duchenne muscular dystrophy. Neurology.

[6] Sussman M. (2002). Duchenne muscular dystrophy. J. Am. Acad. Orthop. Surg..

[7] Falzarano M.S., Scotton C., Passarelli C., Ferlini A. (2015). Duchenne Muscular Dystrophy: From Diagnosis to Therapy. Molecules.

[8] Verhaart I.E.C., Aartsma-Rus A. (2019). Therapeutic developments for Duchenne muscular dystrophy. Nat. Rev. Neurol..

[9] McPherron A.C., Lawler A.M., Lee S.J. (1997). Regulation of skeletal muscle mass in mice by a new TGF-beta superfamily member. Nature.

[10] McPherron A.C., Lee S.-J. (1997). Double muscling in cattle due to mutations in the myostatin gene. Proc. Natl. Acad. Sci. USA.

[11] Schuelke M., Wagner Kathryn R., Stolz L.E., Hübner C., Riebel T., Kömen W., Braun T., Tobin J.F., Lee S.-J. (2004). Myostatin mutation associated with gross muscle hypertrophy in a child. N. Engl. J. Med..

[12] Ashiuchi M., Soda K., Misono H. (1999). A poly-gamma-glutamate synthetic system of Bacillus subtilis IFO 3336: Gene cloning and biochemical analysis of poly-gamma-glutamate produced by Escherichia coli clone cells. Biochem. Biophys. Res. Commun..

[13] Ashiuchi M., Nawa C., Kamei T., Song J.J., Hong S.P., Sung M.H., Soda K., Misono H. (2001). Physiological and biochemical characteristics of poly gamma-glutamate synthetase complex of Bacillus subtilis. Eur. J. Biochem..

[14] Hou X.-L., Yu L.-Y., Liu J., Wang G.-H. (2007). Surface-displayed porcine epidemic diarrhea viral (PEDV) antigens on lactic acid bacteria. Vaccine.

[15] Lee J.-S., Poo H., Han D.P., Hong S.-P., Kim K., Cho M.W., Kim E., Sung M.-H., Kim C.-J. (2006). Mucosal Immunization with Surface-Displayed Severe Acute Respiratory Syndrome Coronavirus Spike Protein on *Lactobacillus casei* Induces Neutralizing Antibodies in Mice. J. Virol..

[16] Poo H., Pyo H.M., Lee T.Y., Yoon S.W., Lee J.S., Kim C.J., Sung M.H., Lee S.H. (2006). Oral administration of human papillomavirus type 16 E7 displayed on Lactobacillus casei induces E7-specific antitumor effects in C57/BL6 mice. Int. J. Cancer.

[17] Ventura M., Jankovic I., Walker D.C., Pridmore R.D., Zink R. (2002). Identification and Characterization of Novel Surface Proteins in *Lactobacillus johnsonii* and *Lactobacillus gasseri*. Appl. Environ. Microbiol..

[18] Xu L.C. (2007). Effects of Avian Myostatin and Synthetic Peptides Expressed in Prokaryotics on Animal Muscle Growth. Ph.D. Thesis.

[19] Bogdanovich S., Krag T., Barton E.R., Morris L.D., Whittemore L.-A., Ahima R.S., Khurana T.S. (2002). Functional improvement of dystrophic muscle by myostatin blockade. Nature.

[20] Qiao C., Li J., Jiang J., Zhu X., Wang B., Xiao X. (2008). Myostatin propeptide gene delivery by adeno-associated virus serotype 8 vectors enhances muscle growth and ameliorates dystrophic phenotypes in mdx mice. Hum. Gene. Ther..

[21] Wagner K.R., Fleckenstein J.L., Amato A.A., Barohn R.J., Bushby K., Escolar D.M., Flanigan K.M., Pestronk A., Tawil R., Wolfe G.I. (2008). A phase I/IItrial of MYO-029 in adult subjects with muscular dystrophy. Ann. Neurol..

[22] Wagner K.R. (2020). The elusive promise of myostatin inhibition for muscular dystrophy. Curr. Opin. Neurol..

[23] Ji S., Losinski Rl Fau-Cornelius S.G., Cornelius Sg Fau-Frank G.R., Frank Gr Fau-Willis G.M., Willis Gm Fau-Gerrard D.E., Gerrard De Fau-Depreux F.F., Depreux Ff Fau-Spurlock M.E., Spurlock M.E. (1998). Myostatin expression in porcine tissues: Tissue specificity and developmental and postnatal regulation. Am J Physiol..

[24] Lee J.-S., Shin K.-S., Pan J.-G., Kim C.-J. (2000). Surface-displayed viral antigens on Salmonella carrier vaccine. Nat. Biotechnol..

[25] Xin K.-Q., Hoshino Y., Toda Y., Igimi S., Kojima Y., Jounai N., Ohba K., Kushiro A., Kiwaki M., Hamajima K. (2003). Immunogenicity and protective efficacy of orally administered recombinant Lactococcus lactis expressing surface-bound HIV Env. Blood.

[26] Patel K., Amthor H. (2005). The function of Myostatin and strategies of Myostatin blockade—new hope for therapies aimed at promoting growth of skeletal muscle. Neuromuscul. Disord..

[27] Zammit P.S., Partridge T.A. (2002). Sizing up muscular dystrophy. Nat. Med..

[28] Barton E.R., Morris L., Musaro A., Rosenthal N., Sweeney H.L. (2002). Muscle-specific expression of insulin-like growth factor I counters muscle decline in mdx mice. J. Cell Biol..

[29] Fox H., Millington L., Mahabeer I., van Ruiten H. (2020). Duchenne muscular dystrophy. BMJ.

[30] Mariot V., Joubert R., Hourdé C., Féasson L., Hanna M., Muntoni F., Maisonobe T., Servais L., Bogni C., Le Panse R. (2017). Downregulation of myostatin pathway in neuromuscular diseases may explain challenges of anti-myostatin therapeutic approaches. Nat. Commun..

[31] Campbell C., Jacob P. (2003). Deflazacort for the treatment of Duchenne Dystrophy: A systematic review. BMC Neurol..

[32] Hanna M.G., Badrising U.A., Benveniste O., Lloyd T.E., Needham M., Chinoy H., Aoki M., Machado P.M., Liang C., Reardon K.A. (2019). Safety and efficacy of intravenous bimagrumab in inclusion body myositis (RESILIENT): A randomised, double-blind, placebo-controlled phase 2b trial. Lancet Neurol..

[33] Dumonceaux J., Marie S., Beley C., Trollet C., Vignaud A., Ferry A., Butler-Browne G., Garcia L. (2010). Combination of Myostatin Pathway Interference and Dystrophin Rescue Enhances Tetanic and Specific Force in Dystrophic mdx Mice. Mol. Ther..

[34] Lu-Nguyen N., Ferry A., Schnell F.J., Hanson G.J., Popplewell L., Dickson G., Malerba A. (2019). Functional muscle recovery following dystrophin and myostatin exon splice modulation in aged mdx mice. Hum. Mol. Genet..

[35] Lu-Nguyen N.B., Jarmin S.A., Saleh A.F., Popplewell L., Gait M.J., Dickson G. (2015). Combination Antisense Treatment for Destructive Exon Skipping of Myostatin and Open Reading Frame Rescue of Dystrophin in Neonatal mdx Mice. Mol. Ther..

[36] Zhou H., Meng J., Malerba A., Catapano F., Sintusek P., Jarmin S., Feng L., Lu-Nguyen N., Sun L., Mariot V. (2020). Myostatin inhibition in combination with antisense oligonucleotide therapy improves outcomes in spinal muscular atrophy. J. Cachex Sarcopenia Muscle.

[37] Verzola D., Barisione C., Picciotto D., Garibotto G., Koppe L. (2019). Emerging role of myostatin and its inhibition in the setting of chronic kidney disease. Kidney Int..

[38] White T.A., Lebrasseur N.K. (2014). Myostatin and Sarcopenia: Opportunities and Challenges—A Mini-Review. Gerontology.

