# Supplementary material for: A New Method of Myostatin Inhibition in Mice via Oral Administration of Lactobacillus casei Expressing Modified Myostatin Protein, BLS-M22
Source: Int J Mol Sci. 2022 Aug 13;23(16):9059. doi: 10.3390/ijms23169059 (PMC9409196; doi:10.3390/ijms23169059)
Supplement: Supplementary file 1 [file ijms-23-09059-s001.zip › ijms-1818620-supplementary.pdf]

Figure S1. Persistent anti-myostatin effect of BLS-M22 with dose-dependent manners

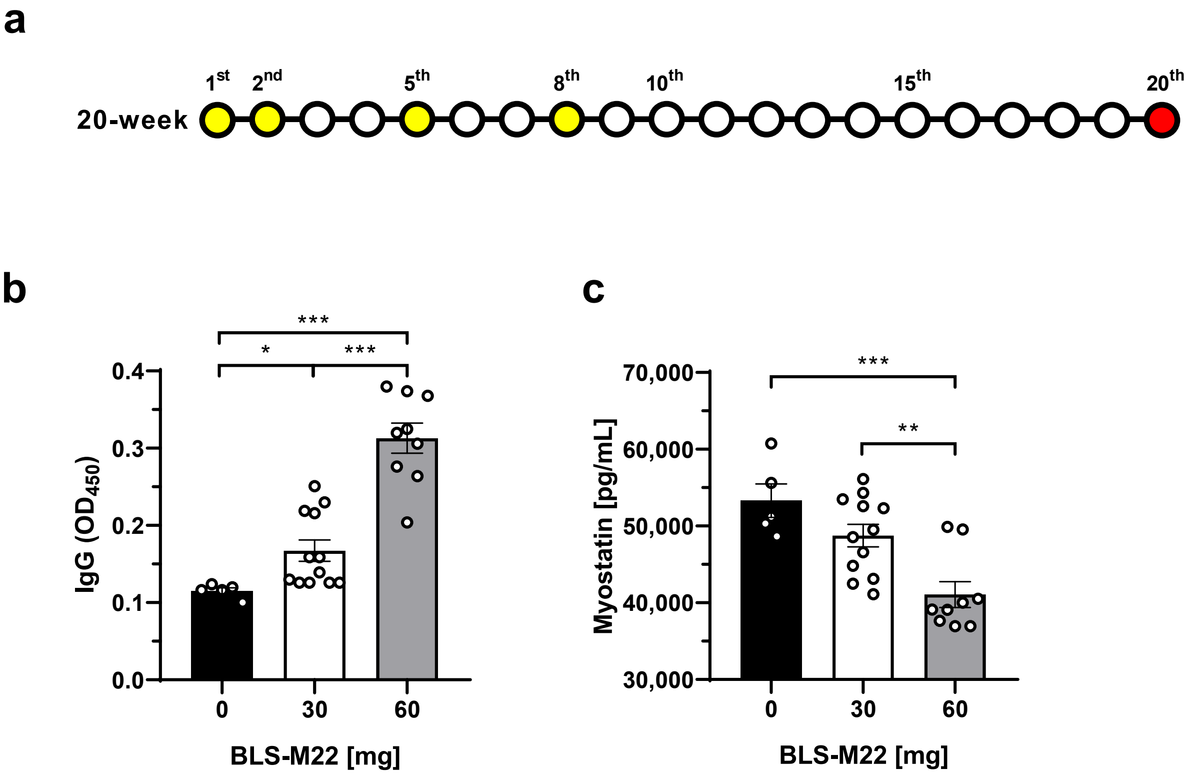

(a) Schematic diagram of BLS-M22 administration. The week where *mdx* mouse were administered BLS-M22 via oral gavage using Zonde needle was indicated in yellow circle. Sacrifice time point were in red. (b) Serum anti-myostatin IgG and (c) the serum concentration of myostatin proteins were measured. All data were represented as mean ± S.E.M. *$^*p < 0.05$, $^{**}p < 0.01$, and $^{***}p < 0.001$.*
